# Modelled health benefits of a sugar-sweetened beverage tax across different socioeconomic groups in Australia: A cost-effectiveness and equity analysis

**DOI:** 10.1371/journal.pmed.1002326

**Published:** 2017-06-27

**Authors:** Anita Lal, Ana Maria Mantilla-Herrera, Lennert Veerman, Kathryn Backholer, Gary Sacks, Marjory Moodie, Mohammad Siahpush, Rob Carter, Anna Peeters

**Affiliations:** 1 Centre for Population Health Research, School of Health and Social Development, Deakin University, Geelong, Victoria, Australia; 2 School of Public Health, University of Queensland, Brisbane, Queensland, Australia; 3 Cancer Council NSW, Woolloomooloo, New South Wales, Australia; 4 Department of Health Promotion, Social & Behavioral Health, College of Public Health, University of Nebraska Medical Center, Omaha, Nebraska, United States of America; Stanford University, UNITED STATES

## Abstract

**Background:**

A sugar-sweetened beverage (SSB) tax in Mexico has been effective in reducing consumption of SSBs, with larger decreases for low-income households. The health and financial effects across socioeconomic groups are important considerations for policy-makers. From a societal perspective, we assessed the potential cost-effectiveness, health gains, and financial impacts by socioeconomic position (SEP) of a 20% SSB tax for Australia.

**Methods and findings:**

Australia-specific price elasticities were used to predict decreases in SSB consumption for each Socio-Economic Indexes for Areas (SEIFA) quintile. Changes in body mass index (BMI) were based on SSB consumption, BMI from the Australian Health Survey 2011–12, and energy balance equations. Markov cohort models were used to estimate the health impact for the Australian population, taking into account obesity-related diseases. Health-adjusted life years (HALYs) gained, healthcare costs saved, and out-of-pocket costs were estimated for each SEIFA quintile. Loss of economic welfare was calculated as the amount of deadweight loss in excess of taxation revenue. A 20% SSB tax would lead to HALY gains of 175,300 (95% CI: 68,700; 277,800) and healthcare cost savings of AU$1,733 million (m) (95% CI: $650m; $2,744m) over the lifetime of the population, with 49.5% of the total health gains accruing to the 2 lowest quintiles. We estimated the increase in annual expenditure on SSBs to be AU$35.40/capita (0.54% of expenditure on food and non-alcoholic drinks) in the lowest SEIFA quintile, a difference of AU$3.80/capita (0.32%) compared to the highest quintile. Annual tax revenue was estimated at AU$642.9m (95% CI: $348.2m; $1,117.2m). The main limitations of this study, as with all simulation models, is that the results represent only the best estimate of a potential effect in the absence of stronger direct evidence.

**Conclusions:**

This study demonstrates that from a 20% tax on SSBs, the most HALYs gained and healthcare costs saved would accrue to the most disadvantaged quintiles in Australia. Whilst those in more disadvantaged areas would pay more SSB tax, the difference between areas is small. The equity of the tax could be further improved if the tax revenue were used to fund initiatives benefiting those with greater disadvantage.

## Abstract 2

Anita Lal and colleagues model for and reveal the potential health benefits and cost savings from a sugar sweetened beverage tax in Australia.

## Author summary

**Why was this study done?:**

- Previous real-world evaluations of a sugar-sweetened beverage (SSB) tax showed that the SSB tax led to a reduction of SSB purchases for the total population, with larger effects for lower-income households.
- It was unknown what the healthcare cost savings, health gains, and financial impacts of an SSB tax would be for different income groups, in Australia or internationally.

**What did the researchers do and find?:**

- We modelled the effect of a 20% SSB tax in Australia on life expectancy and health-adjusted life years before and after implementation of the tax, across quintiles of area-level socioeconomic deprivation.
- Our model predicts that the greatest health gains would accrue to the 2 lowest quintiles (most disadvantaged), leading to the highest healthcare cost savings in these quintiles.
- We estimate the increase in annual expenditure on SSBs to be AU$35 per capita in the lowest quintile, a difference of less than $5 compared to the highest quintile.
- Annual tax revenue was estimated at over AU$640 million.

**What do these findings mean?:**

- A 20% SSB tax in Australia is likely to decrease SSB purchase and consumption, leading to significant health gains and healthcare expenditure savings across all quintiles of socioeconomic deprivation.
- The tax would generate considerable yearly revenue, which the government could use to further improve the health of the most disadvantaged.
- As with all simulation models, the model results represent the best estimate of a potential effect in the absence of stronger direct evidence.

## Introduction

In high-income countries, obesity is more common in the most disadvantaged groups [1]. Reducing inequalities in health between advantaged and disadvantaged groups is an important objective of public health policy [2]. The evidence of the association between sugar-sweetened beverage (SSB) intake and increased energy intake, leading to weight gain and obesity, is compelling [3,4]. Obesity is a strong risk factor for diabetes, cardiovascular disease, some cancers, osteoarthritis, and hypertension [5–7]. Individuals from lower socioeconomic groups have been found to consume more SSBs [8,9]. The prevalence of obesity-related comorbidities is also higher in lower socioeconomic groups.

A tax on SSBs is considered to be an important component of the set of recommended policy approaches to address population obesity [10–12]. Price influences SSB purchase [13], which in turn may reduce the rate of obesity [14]. There is an economic rationale for taxes when consumption results in negative externalities. In Australia, the diseases caused by obesity were estimated to cost tax payers AU$5.3 billion in healthcare costs, forgone tax, and welfare payments in 2014/2015 [15]. The economic rationale for an SSB tax essentially rests on the notion of ‘internalising the externality’ within the purchase price.

There is evidence that people with lower incomes are more sensitive to price increases [16] and are therefore more likely to change their purchasing behaviour in response to price changes. In Mexico, an evaluation of an SSB tax of approximately 10% introduced in 2014 showed a reduction in purchases of taxed beverages for the total population, with an even larger effect for lower-income households [17,18].

The financial impact of an SSB tax for different socioeconomic position (SEP) groups has been examined in terms of the predicted tax burden to individuals and households. A recent systematic review describing the financial burden of an SSB tax across different SEP groups identified 5 studies, which found that the tax would be financially regressive, but with small differences of approximately US$5 between high- and low-income households; the average additional tax paid per household as a result of the SSB tax would be less than US$30 annually across all groups [19]. Previous Australian research has predicted that an SSB tax would lead to cost savings in the health sector [20,21]. But the effect on overall healthcare cost savings and the health gains in health-adjusted life years (HALYs) across SEP groups have rarely been previously examined. The overall financial impact on individuals includes the potential healthcare costs saved by individuals, and this also has seldom been previously estimated across SEP groups. A rate of 20% is the most commonly advocated tax by public health experts [22]. South Africa and the UK have recently proposed taxes of this magnitude [23]. The main aim of this paper, therefore, is to examine the health and financial impacts of a 20% SSB sales tax for Australia across socioeconomic groups by comprehensively integrating distributional aspects into the cost-effectiveness analysis.

This study expands on previous studies in a number of ways. First, the cost-effectiveness of a 20% SSB tax for Australia by SEP subgroup was estimated, including a wide range of SSBs and substitute beverages, with a focus on the quantity and distribution of health gains in HALYs according to an area-based measure of socioeconomic disadvantage, Socio-Economic Indexes for Areas (SEIFA). Second, the distribution of financial impacts to individuals across different SEP groups was examined, in terms of out-of-pocket costs incurred from the tax and healthcare costs saved. Third, the overall economic impact of the tax was examined in terms of the balance of effects for the health sector and the general economy.

## Methods

### Specification of the tax

A 20% sales tax on SSBs in Australia was modelled. SSBs included soft drinks (pop, soda); flavoured water; sports, energy, and fruit drinks; and cordials (concentrates) containing added sugar. It was assumed that the full amount of the tax would be passed on to the consumer.

### Overview

The model estimated the differences in life expectancy and HALYs pre- and post- implementation of the tax. These differences were based on predicted variations in 9 diseases caused by obesity. Changes to body mass index (BMI) were modelled based on projected changes in SSB consumption.

**Fig 1:**
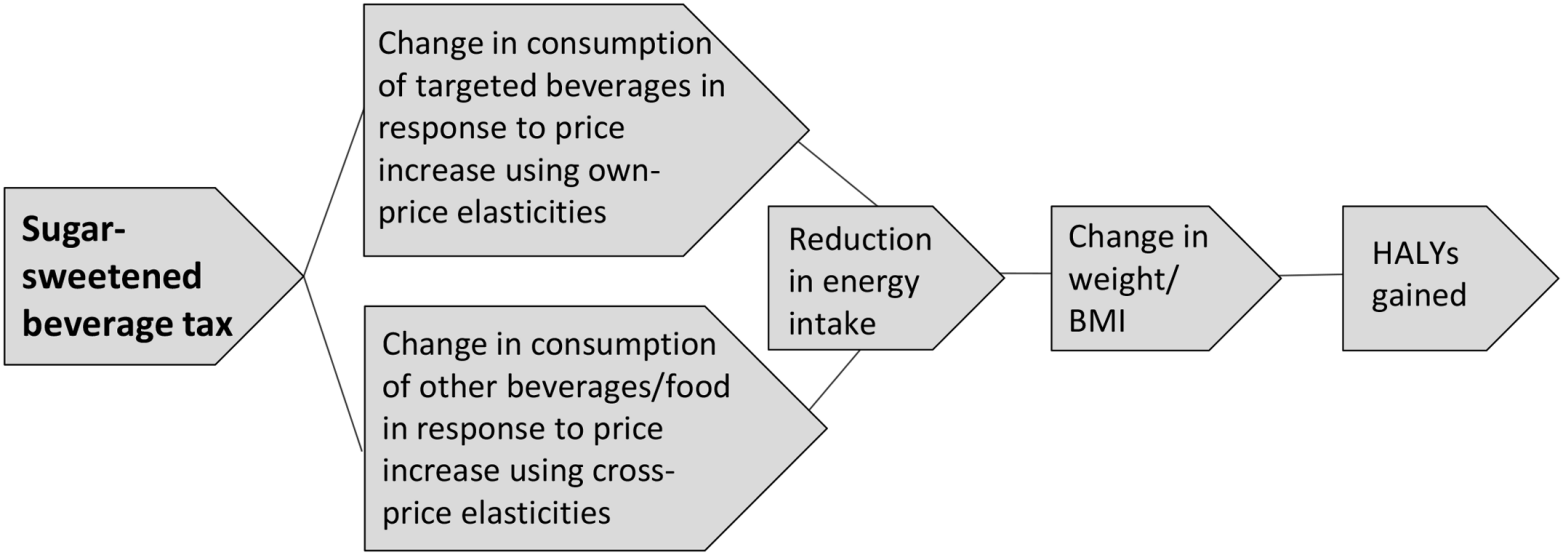
Logic pathway for modelling the health effects of a sugar-sweetened beverage tax. BMI, body mass index; HALY, health-adjusted life year.

The Australian population aged 2–100 years was modelled over a lifetime, with covariates based on the Australian Health Survey (AHS) 2011–12 [24] and disease epidemiology based on a study of the US burden of diseases, injuries, and risk factors in 2010 [25]. The analysis has 2 parts: (1) a whole population analysis and (2) analyses by SEIFA quintile. Fig 1 illustrates the logic pathway of an SSB tax, identifying the steps involved in measuring the expected impact of the tax from an obesity perspective.

### Assessment of benefit

#### Effect of the tax on body weight

For the whole population analysis, baseline intake of drinks was based on data from the AHS 2011–12 [24]. Dietary data were collected based on 24-hour dietary recall on 2 separate days. Mean daily intake of each category of SSB and substitutes of tea, coffee, milk, and 100% fruit juice were extracted from the survey using Stata 14 [26]. Mean intake was calculated by sex, in 5-year age groups.

For the SEP subgroup analyses, individuals in the survey were categorised by SEIFA Index of Relative Socio-Economic Disadvantage (IRSD) deciles, which were converted to quintiles. The SEIFA quintiles represent groups of individuals who live in similarly ranked areas, based on a range of information such as the income, qualifications, and occupation skills of the area residents [27]. Mean SSB intake for the quintiles was not extracted by age group as the groups were too small. However, an age multiplier was applied based on proportions of intake by age from the total population (see S1 Table for mean intake).

The change in intake of SSBs associated with a 20% tax was based on an Australian study that derived own-price elasticities and cross-price elasticities from Australian household supermarket purchases and a mathematical demand system model [28]. Own-price elasticities represent the proportional change in SSB purchases in response to a change in SSB price. Cross-price elasticities represent the proportional change in purchases of other substitute drinks (such as coffee) in response to a change in SSB price. We applied the price elasticities derived for the low- and high-income households, respectively, to the consumption of SSBs in the lowest and highest SEIFA quintile [28]. The price elasticities for the middle-income households were applied to SEIFA quintile 3. The price elasticities for quintiles 2 and 4 were interpolated using linear trends estimation from quintiles 1, 3, and 5 in Excel (see S2 Table). The reductions in quantities of SSBs consumed and increases in substitute drinks consumed were converted to kilojoule equivalents using nutrient tables for Australia (NUTTAB, 2010) [29].

Estimated changes in body weight for adults were calculated based on published relationships between changes in energy expenditure and body weight at the population level. Changes in body weight for adults were based on a change of 100 kilojoules per day equalling a 1-kg change in weight, taking 3 years to achieve the total weight change [30]. The following formulas were used for children [31]:

Boys: kilojoules per day per kilogram = 68−2.5*age*4.184 (1)

Girls:kilojoules per day per kilogram = 62−2.2*age*4.184 (2)

These changes in weight were converted to changes in BMI using average Australian height and weight by gender and single-year age groups up to 19 years and 5-year age groups thereafter from the AHS 2011–12 [24].

#### Modelling health outcomes

We used a recently developed model (CRE-Obesity model) to estimate how a change in the distribution of the prevalence of overweight and obesity caused by an intervention impacts the epidemiology of several obesity-related diseases, which in turn influence the total HALYs in the population. This model was based on the Assessing Cost-Effectiveness in Prevention (ACE-Prevention) obesity model [32,33], which has previously been used to model the cost-effectiveness of several obesity interventions [34–36]. We extended the model by considering the incremental costs and benefits by SEIFA quintile, as well as by including children and adolescents.

The model uses a multi-state, multiple cohort life table approach to translate changes in mean BMI following an intervention into corresponding HALYs gained. Potential impact fractions were used to quantify the proportional reduction in disease incidence that would occur if a population were subject to a counterfactual exposure to a risk factor because of an intervention [37]. Disease-specific mortality and morbidity were then combined with all other causes of mortality and morbidity from the population to estimate the total morbidity and mortality in the total population. The diseases were diabetes mellitus, ischemic heart disease, stroke, hypertensive heart disease, colorectal cancer, breast cancer, endometrial cancer, kidney cancer, and osteoarthritis of the knee and hip.

The CRE-Obesity model calculates incremental HALYs, incremental costs, and cost-effectiveness ratios. Using the SEIFA IRSD, we created quintile-specific sub-models by substituting key input parameters with SEIFA-quintile-specific data. These parameters included disease incidence [38–40], mortality rate [41], BMI distribution [42], and population number [43]. We modelled the SSB tax on SEIFA groups as a population-based intervention—that is, the lifetime health and cost effects from the tax altered the distribution of BMI among all ages (2–100 years). For individuals aged 2–19 years, who were not modelled as experiencing the included diseases, the disability related to obesity itself was quantified, using the health-related quality of life lost due to obesity before and after the intervention, based on the difference between quality-adjusted life year (QALY) weights [44]. Thus, HALYs gained due to an intervention represent the number of years lived in full health gained, adjusted for morbidity of obesity-related diseases, and obesity as a whole in the population aged less than 20 years.

### Assessment of costs

#### Intervention costs

Intervention costs were assessed from both a government and industry perspective over the lifetime of the population. In the absence of Australian data on the administration and compliance costs of implementing a soft drink tax, costing methods from a US study of 2 states operating an excise tax were converted to the equivalent Australian costs [45]. Estimates of legislation costs of tobacco plain packaging in terms of establishment, implementation, ongoing compliance, and administration costs for the Australian Department of Health, at AU$12.69 million (m) over 10 years, were used as a benchmark [46]. Administration costs to the beverage industry were assumed to be equal to the costs to government, based on sales tax evidence in the US [47].

A framework for costing new public health legislation was used to estimate the cost of passing legislation in the Australian parliament [47], with slight adjustments for the Australian context. Briefly, it includes parliamentarians’ time, annual expenses for the House of Representatives and the Senate, legislation drafting, and publication and policy advice. As we could not identify costs for policy advice in Australia, we used the equivalent Australian dollar costs from New Zealand (NZ) (see S3 Table).

#### Healthcare costs

Treatment costs were based on Disease Costs and Impact Study (DCIS) 2001 data from the Australian Institute of Health and Welfare (AIHW) [48], inflated to 2010 prices using AIHW health price inflation values [49]. Costs included hospital services, out-of-hospital medical services, pharmaceuticals, and health professionals. Healthcare expenditures saved were estimated based on the predicted reduction in mortality and morbidity from the 9 diseases.

#### Out-of-pocket healthcare costs

Out-of-pocket healthcare costs are healthcare costs paid for by individuals and include pharmaceuticals, medical services, practitioners, aids and appliances, and hospital costs. Out-of-pocket healthcare costs by SEIFA quintile were based on the percentage of individuals’ overall expenditure used for total healthcare expenditure, reported by the AIHW in 2010 as 17.4% [49]. Proportions of mean household annual healthcare expenditure and total household expenditure, together with ‘equivalised’ disposable income by quintile from the Australian Bureau of Statistics Household Expenditure Survey 2010 [50], were used to calculate quintile-specific out-of-pocket healthcare costs as a percentage of overall expenditure. Equivalisation is a technique in economics whereby members of a household receive different weightings. Total household income is then divided by the sum of the weightings to yield a representative income.

#### Deadweight loss (loss in economic welfare)

Deadweight loss (DWL) is an economic term used to describe the net loss in total economic welfare that can be attributed to the introduction of a new tax or tax increase. The tax drives a price increase that leads to a fall in demand; this in turn involves reduced benefits flowing to both consumers and producers. As a result, there is a reduction in both consumer surplus (the difference between the value a consumer places on a product and the price paid) and producer surplus (the price minus the economic cost of producing the product) (refer to S1 Fig for additional detail). This loss in economic welfare is calculated for each SSB category for each quintile and for the population using the following formula:

DWL =0.5 * (P2—P1)*(Q1—Q2) (3)

where P1 is the original price of the SSBs, P2 is the new price of the SSBs, Q1 is the original quantity demanded of the SSBs, and Q2 is the new quantity demanded of the SSBs.

The total loss of economic welfare is thus the amount of DWL in excess of the taxation revenue collected by the government. There are also behavioural responses associated with these inherently dynamic impacts that are not fully captured in this formula—desirably, industry realigns to healthier products, consumers realign to healthier purchases, and the tax revenue can be utilised for welfare-enhancing initiatives.

#### Out-of-pocket tax costs

Predicted tax paid per person due to the introduction of a 20% SSB tax was calculated as the post-tax mean quantity demanded of each category of SSB consumed multiplied by 20% of the current retail price. This assumes that the full burden of the tax is borne by the consumer. We used prices sourced from a large Australian supermarket website (Coles; http://www.colesonline.com.au). An average price per litre was taken from a range of sizes and brands. Mean annual expenditure on food and non-alcoholic drinks by ‘equivalised’ disposable income quintile from the Australian Household Expenditure Survey 2010 [50] was used to calculate percentage of annual expenditure on food and non-alcoholic drinks.

#### Tax revenue

The tax revenue predicted to be received by the government was calculated by multiplying the per person out-of-pocket tax cost by the number of people in each population group.

### Cost-effectiveness analysis

Table 1 outlines the general methodology of the economic evaluation. The intervention was assumed to be operating in ‘steady state’ (i.e., running at its full effectiveness potential) and was measured against current practice. Establishment costs were included in the cost of the intervention. The additional costs and the associated health benefits (HALYs) resulting from the tax were used to calculate incremental cost-effectiveness ratios (ICERs), defined as the difference in net costs of the tax compared to no tax, divided by the difference in net HALYs.

**Table 1:** General economic evaluation methods.

Parameter	Method
Perspective	Societal perspective with costs split into health sector, other government, industry, and private
Economic framework	Cost—utility analysis by subgroup using Socio-Economic Indexes for Areas (SEIFA) Index of Relative Socio-Economic Disadvantage quintiles, as well as for the total population
Monetary unit of measurement	Australian dollars
Base year	2010
Unit of measurement of outcomes	Health-adjusted life years (HALYs) include disability-adjusted life years (DALYs) for diseases prevented for adults and a quality of life adjustment for disability attributed to obesity itself for those aged under 20 years (using quality-adjusted life year [QALY] weights from the literature)
Comparator	Current practice
Discount rate	3%, as recommended by a consensus panel of US health economists and which approximates the real rate of return on long-term government bonds [51]
Time frame	Lifetime

### Uncertainty and sensitivity analysis

The impact of uncertainty around input values on the main outcome measures was estimated by Monte Carlo simulations (Table 2). Means and 95% confidence intervals for BMI effects on HALYs and intervention costs were reported based on 2,000 iterations using Ersatz version 1.3 software [52].

**Table 2:** Key model parameters.

Parameter	Value	95% confidence interval	Source and modelling parameters
**Change in consumption and weight**			
Daily intake of SSB and substitutes	See S1 Table	See S1 Table	Normal distribution of gender- and age-specific means from the Australian Health Survey 2011–12
Own- and cross-price elasticities of demand	See S1 Table	Not specified	Sharma et al. [28]; standard errors calculated based on *Z* score with alpha 0.1, 2-tailed (probability 0.05)
Change in consumption of beverages to change in weight	100-kJ/day change = 1-kg change in weight		Hall et al. [30]
**Cost of implementing an SSB tax**			
Cost of passing legislation in parliament	$1,090,000	$948,000–$1,251,000	Gamma distribution, SE $77,497
Administration and compliance time costs per million people (FTE, government and industry)	0.32	0.10–0.54	Long et al. [45]
Field audit time costs per million people per year (FTE, government and industry)	0.30	0.24–0.35	Samples drawn from beta distribution, Long et al. [45]
Field audit direct costs per million people per year (government and industry)	$10,300	$13,800–$17,200	Samples drawn from a gamma distribution (5th percentile $10,300, 95th percentile $17,200) based on an estimate of field audit direct costs [45]
Accountant yearly salary (government)	$84,900	$78,500–$91,250	Gamma distribution, SE $3,250; ABS mean salary for accountants and auditors, code 2211, 2212*; assumes 14% non-salary benefits [53]
Accountant yearly salary (industry)	$98,400	$88,500–$108,300	Gamma distribution, SE $5,044; ABS mean salary for accountants and auditors, code 2211, 2212*; assumes 14% non-salary benefits [53]

All costs are in Australian dollars. ABS, Australian Bureau of Statistics; FTE, full-time equivalent; SSB, sugar-sweetened beverage.

*Includes superannuation and payroll tax.

We performed several sensitivity analyses. We performed one-way sensitivity analyses to explore the effect of including flavoured milk in the SSBs. As the price elasticity for flavoured milk was not available, we assumed the same price elasticity as for soft drinks. We also tested an SSB tax rate of 30% and a 50% pass-through of the 20% tax. Another mechanism for implementing a tax—a 50¢ per litre volumetric tax was also tested. This is in line with alcoholic beverages in Australia, which are taxed per litre of alcohol. A tax of 50¢ per litre is an average 17% increase in price across all SSB categories.

### Health equity analysis

A concentration index quantifies the degree of socioeconomic inequality in a specific health variable. Concentration indices were calculated for each tax scenario (sensitivity analysis) to quantify the degree to which HALYs gained are concentrated in disadvantaged groups. The index takes a negative value when HALY gains are greater amongst the most disadvantaged, and a positive value when HALY gains are greater amongst the least disadvantaged. The concentration index was calculated for each tax scenario using the following formula [54]:

$$C=\frac{2}{u}\sum_{t-1}^{T}f_{t}u_{t}R_{t}-1$$ (4)

where *u*_*t*_ is the mean number of HALYs of the *t*th SEIFA group, *f*_*t*_ is its population share, and *R*_*t*_ is the fractional rank of SEIFA group *t*.

## Results

**Fig 2:**
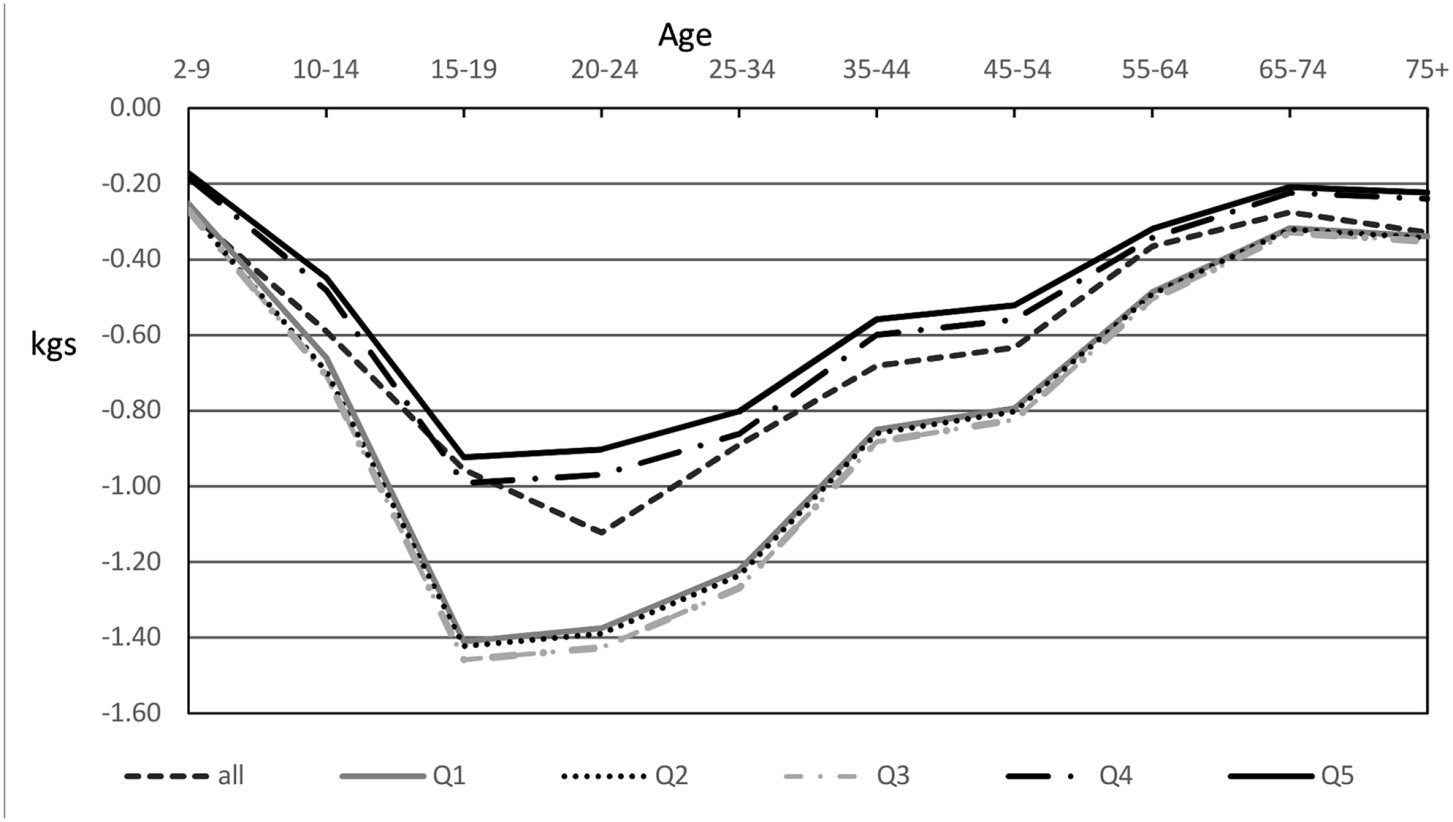
Modelled mean weight decreases in men after introduction of a 20% sugar-sweetened beverage tax by quintile. Q1 is the most disadvantaged quartile, and Q5 is the least disadvantaged quartile.

**Fig 3:**
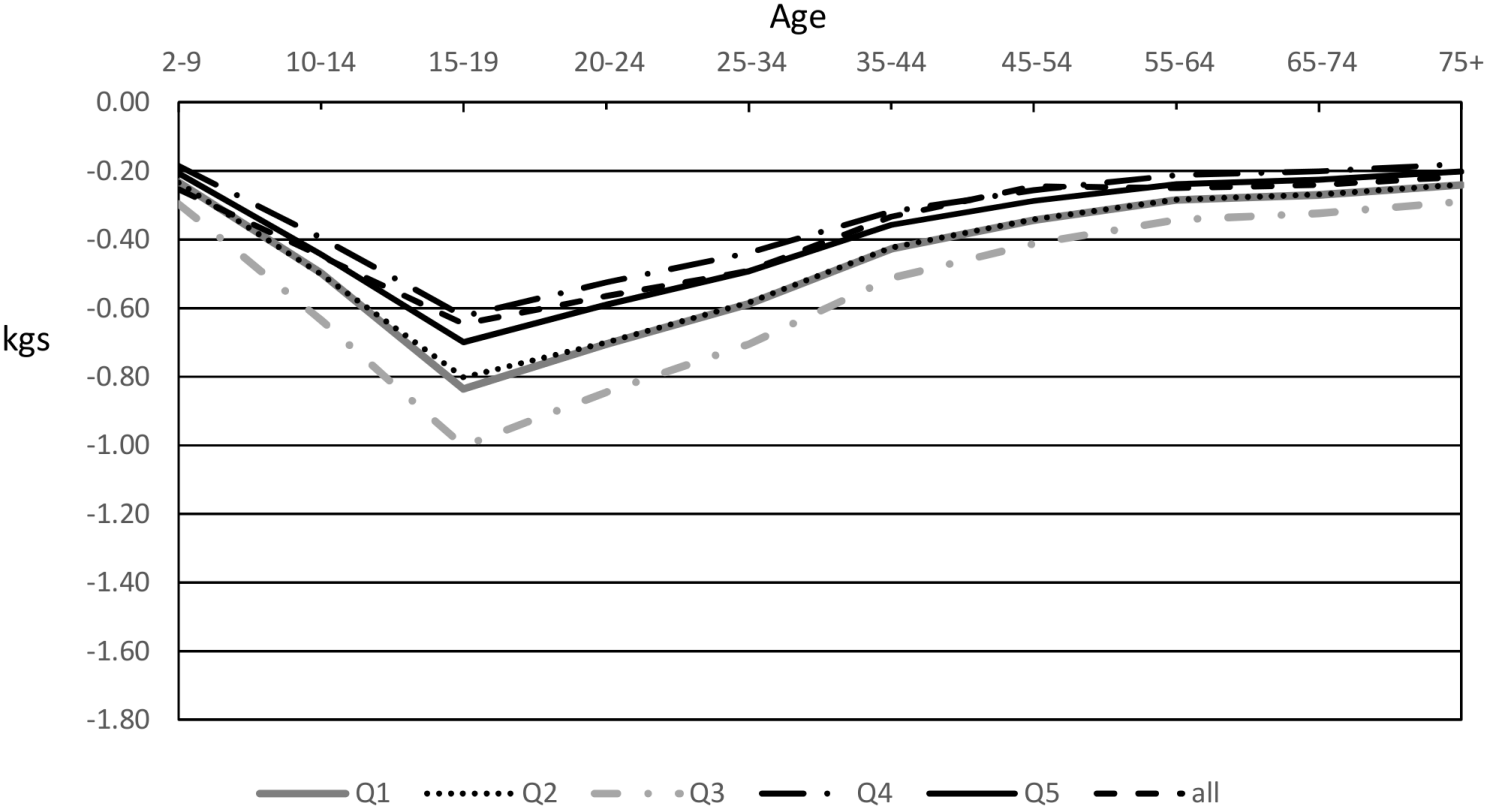
Modelled mean weight decreases in women after introduction of a 20% sugar-sweetened beverage tax by quintile. Q1 is the most disadvantaged quartile, and Q5 is the least disadvantaged quartile.

Enacting a 20% SSB sales tax in Australia was estimated to result in greater decreases in weight for the 3 most disadvantaged quintiles than for the 2 least disadvantaged quintiles for both men and women, with larger decreases in men. Quintile 5 (least disadvantaged) had the lowest predicted reductions in weight for men and women (Figs 2 and 3).

As a result of a 20% SSB tax, the Australian population was estimated to gain 175,300 HALYs (95% CI: 68,700; 277,800) and save 111,700 years of life (95% CI: 43,600; 175,800) (Table 3). The HALY gains were highest in the 2 most disadvantaged quintiles, with 49.4% of the total HALYs gained accruing to these quintiles. Quintile 5 had the lowest HALYs gained and years of life saved for men and women.

**Table 3:** Cost-effectiveness results of a 20% sugar-sweetened beverage tax.

	Quintile 1 (most disadvantaged)	Quintile 2	Quintile 3	Quintile 4	Quintile 5	Total population
Total HALYs saved over lifetime (thousands)	52.3 (15.4, 85.2)	49.9 (28.2, 71.5)	48.8 (19.7, 75.3)	31.7 (26.3, 38.8)	27.4 (12.7,42.7)	175.3 (68.7, 277.8)
Total years of life saved over lifetime (thousands)	39.9 (12.2, 65.0)	37.2 (21.3, 52.9)	37.1 (15.4, 57.2)	23.4 (20.0, 27.9)	21.0 (9.9, 32.4)	111.7 (43.6, 175.8)
Total healthcare cost savings over lifetime (millions of dollars)	435.7 (308.1, 564.7)	429.8 (247.1, 606.0)	394.0 (334.9, 461.0)	293.8 (240.8, 358.1)	254.7 (217.5, 296.0)	1,732.9 (650.1, 2,744.0)
Healthcare cost savings per capita over lifetime (dollars)	104 (74, 136)	103 (59, 146)	95 (74, 186)	71 (58, 86)	61 (52, 71)	83 (31,131)
Net cost per HALY saved	Cost saving	Cost saving	Cost saving	Cost saving	Cost saving	Cost saving
Out-of-pocket healthcare costs saved over lifetime (millions of dollars)	33.7 (12.7, 53.4)	58.2 (21.8, 92.2)	55.3 (20.8, 87.6)	65.1 (24.4, 103.1)	89.1 (33.4, 141.1)	301.5 (113.1, 477.5)
Out-of-pocket healthcare costs saved as percent of household expenditure	1.23%	1.43%	1.10%	1.06%	1.11%	1.17%
Out-of-pocket costs of tax (dollars per person, yearly)	35.40 (18.70, 62.80)	31.10 (15.90, 56.10)	34.70 (21.40, 53.90)	28.90 (11.50, 52.70)	31.60 (17.70, 55.90)	31.30 (17.10, 55.40)
Out-of-pocket costs of tax as percent of annual expenditure on food and non-alcoholic drinks	0.54%	0.33%	0.31%	0.24%	0.22%	0.30%

Values in parentheses are 95% CIs; dollar amounts are in Australian dollars. Quintile totals do not add up to the population total as results are based on quintile-specific data and populations. Lifetime costs and health-adjusted life years (HALYs) are discounted at 3%.

The tax was estimated to be cost saving across all intervention scenarios (sensitivity analyses) and all quintiles. Over the lifetime of the population cohort, expected healthcare cost savings were AU$1.73 billion, intervention costs were estimated to be AU$119.6m (95% CI: $91.9m; $162.1m)—approximately $4.8m (95% CI: $3.9m; $6.1m) in the first year and $3.7m (95% CI: $2.8m; $5.0m) in subsequent years, discounted at 3%. For every dollar invested in the first 10 years, the tax would result in AU$17 (95% CI: $9; $19) in healthcare cost savings. The tax revenue generated at the population level was estimated to be AU$642.9m annually (95% CI: $348.2m; $1,117.2m).

For the total population, the out-of-pocket healthcare costs saved were estimated to be AU$299.4m (95% CI: $113.8m; $476.2m). Healthcare cost savings as a percentage of household expenditure by quintile were highest in the most disadvantaged groups. Per capita, the most disadvantaged quintile was estimated to incur the most tax, at an estimated AU$35.40 (95% CI: $18.70; $62.80) per year, or 0.54% of expenditure on food and non-alcoholic drinks.

The tax revenue raised outweighed the DWL for the total Australian population, with an estimated net deadweight impact of +AU$587.9m (95% CI: +$329.2m, +$1,027.6m) per year. The DWL was more than offset by tax revenue across all quintiles, with substantial net gains in each quintile (Table 4).

**Table 4:** Estimated net deadweight impact of a 20% tax on sugar-sweetened beverages.

Population	Deadweight loss	Tax revenue	Net deadweight impact*
Total**	55.0 (19.0, 89.6)	642.9 (348.2, 1,117.2)	+587.9 (+329.2, +1,027.6)
Quintile 1 (most disadvantaged)	17.7 (4.2, 29.2)	147.6 (78.0, 261.7)	+129.8 (+73.8, +232.5)
Quintile 2	12.1 (3.0, 20.4)	129.4 (66.2, 233.5)	+117.3 (+63.2, +213.0)
Quintile 3	15.1 (4.9, 24.4)	127.6 (69.5, 220.0)	+112.5 (+64.6, +197.6)
Quintile 4	10.5 (2.4, 17.8)	120.3 (62.2, 219.2)	+109.8 (+59.8, +201.4)
Quintile 5	6.3 (1.6, 10.9)	131.3 (73.3, 232.0)	+125.0 (+71.7, +221.1)

Values are given in millions of Australian dollars, with 95% CIs in parentheses.

*These values are sometimes shown as negative deadweight losses (double negative), but it is less confusing to show these values as net positive dollar impacts.

**Quintile totals do not add up to the population total as results are based on quintile-specific data and populations.

### Sensitivity analyses

The SSB tax remained cost saving when (1) the pass-through rate was 50%, (2) the rate of the tax was 30%, (3) flavoured milk was included as an SSB, and (4) a volumetric tax was applied at 50¢ per litre (see S4 Table). For each dollar invested in the first 10 years, the resulting healthcare cost savings ranged from $10 to $25 (Table 5).

**Table 5:** Returns on investment in healthcare cost savings and concentration indices of tax scenarios.

Outcome	20% tax	50% pass-through of 20% tax	30% tax	50¢ per litre tax	20% tax includes flavoured milk
Returns on investment in healthcare cost savings in first 10 years (95% CI)	$17 (9; 19)	$10 (8; 11)	$25 (21; 25)	$11 (9; 13)	$20 (16; 21)
Concentration index (standard error)	−0.130 (0.037)	−0.145 (0.024)	−0.140 (0.036)	−0.144 (0.071)	−0.139 (0.044)

Dollar amounts are in Australian dollars.

All tax scenarios have a negative concentration index, indicating that the highest proportion of HALYs gained is amongst the most disadvantaged quintiles. The 50% pass-through of a 20% tax and the 50¢ per litre tax had the largest negative indices, indicating the most equitable scenarios (Table 5).

The 30% tax rate scenario resulted in the largest difference between the lowest and highest quintiles in terms of out-of-pocket costs for the tax; however, this scenario resulted in the largest health gains and healthcare costs saved across the population. Compared to a 20% tax, a 50¢ volumetric tax resulted in smaller health gains across all SEIFA quintiles, due to the level of tax translating to a lower level of price increase across all drink categories.

## Discussion

In our study we estimate that a 20% sales tax on SSBs in Australia would result in the largest number of obesity-related HALYs being averted in the population living in the most disadvantaged SEIFA quintiles, and it follows that the most healthcare cost savings overall would accrue to these groups. The expected out-of-pocket tax expenditure was highest in the most disadvantaged quintile; however, the difference of 0.32% points (less than 10¢) between the lowest and highest quintiles in proportion of household spending on food and non-alcoholic beverages per week was small. Our results indicate that, as a proportion of overall spending, the lowest SEIFA quintiles would have the largest out-of-pocket healthcare cost savings.

The DWLs for each SEIFA quintile, as well as for the whole population, were negative. This indicates that the loss of consumer/producer benefit would be outweighed by the amount of tax collected under our assumptions. The ‘loss in economic welfare’ is often calculated as the dollar amount of DWL in excess of dollar taxation revenue collected by the government. In our analysis there is a substantial net taxation gain suggestive of an improvement in economic welfare (this underlies the rationale for internalisation of negative externalities). But there are also behavioural responses associated with this inherently dynamic interaction that are difficult to model in these formulaic terms—desirably, industry would realign to healthier products and minimise its loss in producer surplus, consumers would realign to healthier purchases and minimise their loss in consumer surplus, and, finally, the tax revenue could be utilised for welfare-enhancing initiatives.

In the United Kingdom, it was considered reasonable to assume a pass-through rate of 100%; however, empirical evidence is mixed. The effect of manufacturers or retailers absorbing part of the tax could decrease the impact of the tax and the resulting health benefits; however, based on our predicted results for a 50% pass-through, the healthcare cost savings would nevertheless be substantial. There could also be an additional ‘halo effect’—a decrease in purchasing of SSBs from the introduction of the tax caused by increased public health awareness.

This research builds on the growing evidence that a tax on SSBs would deliver the largest health gains for the lowest socioeconomic groups. It also reinforces previous findings that the overall amount of tax per capita for a 20% value-added tax is around $30 per year, or less than 60¢ per week, and differences in tax expenditure between the lowest and highest socioeconomic groups are small [19].

The predicted body weight losses in our study are lower than those in Sharma et al.’s study [28], and this is because we took into account age and sex differences. Tax expenditures in our study are higher overall, and this is due to different price assumptions, as well as differing baseline intake of SSBs. The differences in baseline intake can be explained by the differing data collection methods. We used individual survey data recorded over 2 days from the AHS 2011–12, from which we took an average daily intake. These averages are close to the estimates from Euromonitor International of per capita purchases of SSBs in Australia [55]. Our predicted HALYs saved and expected tax revenue are slightly higher than in the previous Australian study that also modelled a 20% tax on SSBs [21], but this to be expected given that we included children in our analysis. Our predicted healthcare cost savings are higher due to a different method for calculating cancer treatment costs, based on the incidence rates rather than prevalence, and all costs have been updated to 2010.

This is possibly the first cost-effectiveness study to include the explicit health and economic outcomes by SEP and the resulting DWLs. It also expands on previous Australian research to include a wider range of SSBs and substitute beverages. We used conservative own-price elasticities that were close to half the value of other published price elasticities for soft drinks [45,56]. They take into account that SSB prices are not fixed, and households might face a quality—quantity tradeoff for each beverage and could opt for cheaper brands if they prefer quantity over quality [28].

When comparing our average changes in adult kilojoule intake per day for the population to a randomised control trial of overweight and obese adults who replaced all caloric SSBs with non-caloric beverages, the tax has approximately 20%–26% of the impact of the results from the trial (49 kcal/day decrease in our model versus 260 kcal/day and 187 kcal/day decreases for overweight and obese individuals, respectively, in the trial) [57]. This proportion of the effect is similar to the average change in own-price elasticities of consumption across all categories of SSBs of approximately 23% for all households as a result of a 20% SSB tax when compared to the trial [28].

As with all simulation models, the model results represent the best estimate of a potential effect in the absence of stronger direct evidence. We used an aggregate area-based indicator of SEP (SEIFA), as we were unable to obtain income-specific input data. We therefore assumed that price elasticities for household income groups were similar for SEIFA groups. There are also inherent limitations of survey data, such as misreporting, which may have affected the baseline intake of SSBs. Cross-price elasticities of food substitutes by SEP were not available, so these were not included [58]. Around 75% of soft drink sales are from supermarkets, and prices may be slightly different to our estimates.

In some instances, we used costing frameworks from the US and NZ in the absence of Australian estimates. NZ costs for policy advice provided by government agencies to parliament related to new laws is likely to be similar to their Australian equivalents, as the legal systems and number of new laws passed in Australia and NZ are similar. Costs to the government for compliance and administration of the introduction of plain packaging of tobacco products in Australia were similar to our estimates [46].

The model does not incorporate the effects of changes in SSB consumption on oral health or indirect costs, such as reduced productivity due to absenteeism and disability, which means that the societal savings from the intervention are likely to be substantially underestimated, especially to those in the most disadvantaged groups. The assumptions for the quality of life lost in children due to obesity are based on the best available evidence, but this evidence is from only particular age groups of school-aged children, and we have assumed the effects are similar for a wider age group.

Dedicating a portion of the substantial revenue generated from SSB taxes to efforts to reduce and prevent obesity among the most disadvantaged populations could be a way to further reduce concerns about the impact of the tax on low SEP groups. Hypothecation of taxes is also effective in generating public support [59]. There is evidence in Australia that earmarking the tax revenue for subsidising healthy food [60], tackling childhood obesity, and supporting children’s sport [61] and health promotion initiatives [62] would raise the public support for such a tax. Future studies could examine where to direct the revenue from an SSB tax for optimal equity, efficiency, and affordability.

Many countries and jurisdictions around the world have committed to an SSB tax, and this analysis shows that a 20% SSB tax is likely to result in a decrease in the purchase and consumption of sugary drinks, leading to significant health gains and healthcare expenditure savings across all quintiles of SEP. The tax would result in considerable yearly revenue that the government could use to reduce the regressive financial impacts, by funding programs to further improve the health of the most disadvantaged. Australia should consider a tax on SSBs as part of a suite of recommended policies to reduce the rates of obesity.

## Supporting information

S1 CHEERS Checklist CHEERS checklist. (PDF)

S1 Data Disease incidence by socioeconomic position. (XLSX)

S1 Fig Deadweight loss of sugar-sweetened beverage tax. (PDF)

S1 Model Sugar-sweetened beverage tax obesity model for Australia. (XLSX)

S2 Model Sugar-sweetened beverage inputs for model. (XLSM)

S1 Table Mean intake of sugar-sweetened beverages in Australia in 2011. (PDF)

S2 Table Impact of a 20% tax on demand for sugar-sweetened beverages by income group and total population. (PDF)

S3 Table Estimating the cost of sugar-sweetened beverage tax legislation. (PDF)

S4 Table Cost-effectiveness results of the sensitivity analyses. (PDF)

## Abbreviations

AHS: Australian Health Survey
AIHW: Australian Institute of Health and Welfare
BMI: body mass index
DWL: deadweight loss
HALY: health-adjusted life year
IRSD: Index of Relative Socio-Economic Disadvantage
m: million
NZ: New Zealand
QALY: quality-adjusted life year
SEIFA: Socio-Economic Indexes for Areas
SEP: socioeconomic position
SSB: sugar-sweetened beverage
