# Correction: Modelled health benefits of a sugar-sweetened beverage tax across different socioeconomic groups in Australia: A cost-effectiveness and equity analysis

**DOI:** 10.1371/journal.pmed.1003310

**Published:** 2020-07-29

**Authors:** Anita Lal, Ana Maria Mantilla-Herrera, Lennert Veerman, Kathryn Backholer, Gary Sacks, Marjory Moodie, Mohammad Siahpush, Rob Carter, Anna Peeters

In supporting information file S4 Table, some table row headers were omitted in the final version of the table, leading to incorrect numbers in the rows affected. For example in the last section ‘50c per litre’, the row heading ‘% expenditure food and non-alcoholic drinks’ is missing.

Please see the corrected S4 Table.

## Supporting information

S4 Table Cost-effectiveness results of the sensitivity analyses. (PDF)
