# Supplementary material for: Modelled health benefits of a sugar-sweetened beverage tax across different socioeconomic groups in Australia: A cost-effectiveness and equity analysis
Source: PLoS Med. 2017 Jun 27;14(6):e1002326. doi: 10.1371/journal.pmed.1002326 (PMC5486958; doi:10.1371/journal.pmed.1002326)
Supplement: S1 Fig — (PDF) [file pmed.1002326.s003.pdf]

S1 Fig. Deadweight loss of a SSB tax

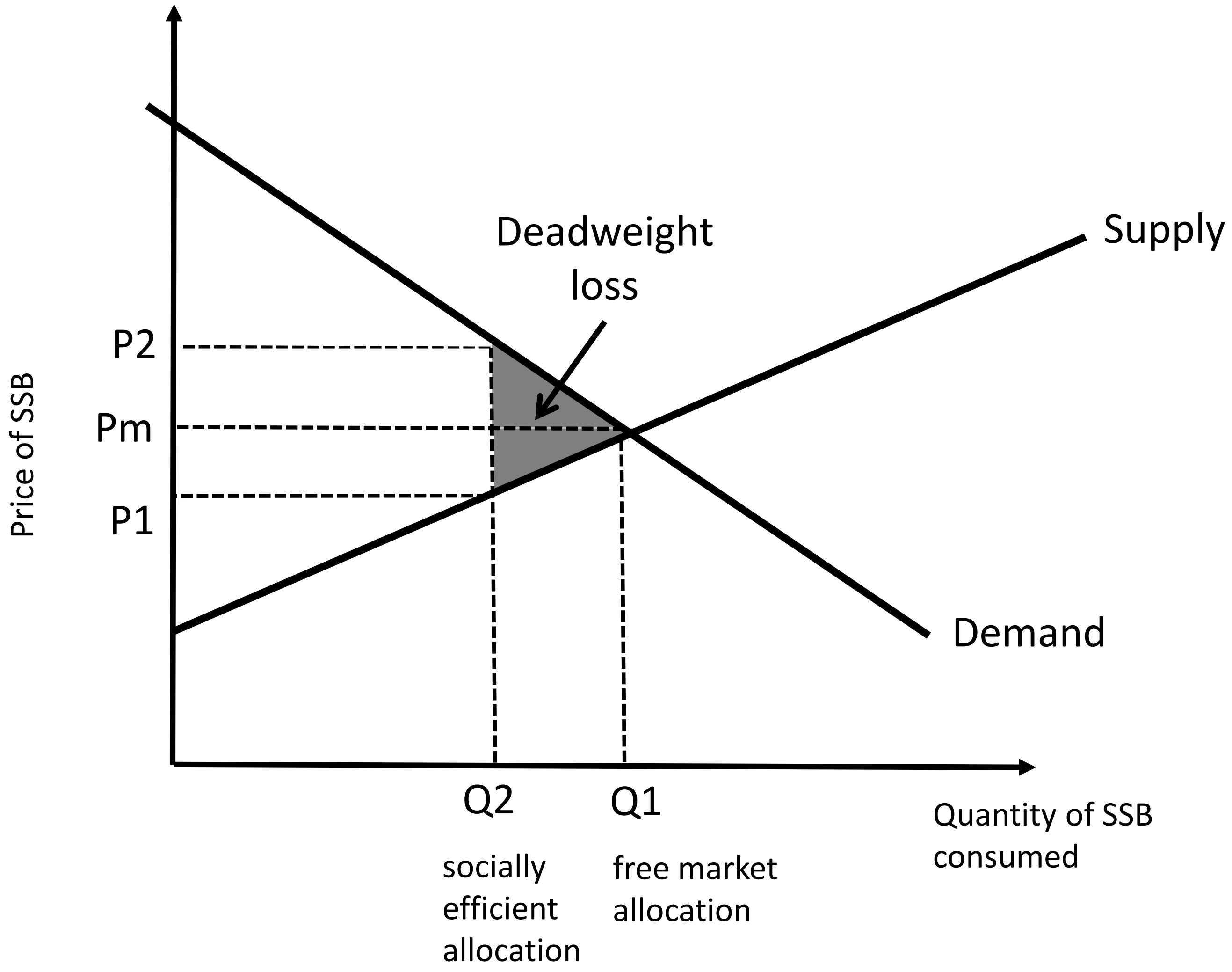

Notes: Pm = market price prior to tax. P1 = price sellers receive, P2= price after tax, Q1 = original quantity demanded, Q2 = quantity demanded after tax.
