# Supplementary material for: Modelled health benefits of a sugar-sweetened beverage tax across different socioeconomic groups in Australia: A cost-effectiveness and equity analysis
Source: PLoS Med. 2017 Jun 27;14(6):e1002326. doi: 10.1371/journal.pmed.1002326 (PMC5486958; doi:10.1371/journal.pmed.1002326)
Supplement: S1 Table — (PDF) [file pmed.1002326.s006.pdf]

S1 Table SSB Intake

	Quintile 1		Quintile 2		Quintile 3		Quintile 4		Quintile 5	
Consumption of SSB	Males 2+	Females 2+	Males 2+	Females 2+	Males 2+	Females 2+	Males 2+	Females 2+	Males 2+	Females 2+
Mean intake carbonated drinks (g/day)	145.0	96.5	143.9	74.1	125.9	73.9	128.0	71.1	110.2	51.1
Relative standard error of estimate (%)	11.7	7.2	10.5	7.2	12.1	5.8	9.6	7.4	11.0	6.0
Lower 95% CI	121.5	82.1	122.9	59.6	101.7	62.2	108.8	56.3	88.2	39.2
Upper 95% CI	168.4	111.0	164.8	88.5	150.1	85.6	147.1	86.0	132.2	63.1
Mean intake sports drinks (g/day)	7.7	7.7	4.4	4.4	8.2	8.2	8.4	8.4	8.2	8.2
Relative standard error of estimate (%)	0.9	0.9	1.3	1.3	1.9	1.9	2.0	2.0	1.5	1.5
Lower 95% CI	6.0	6.0	1.8	1.8	4.5	4.5	4.5	4.5	5.2	5.2
Upper 95% CI	9.5	9.5	7.1	7.1	12.0	12.0	12.3	12.3	11.2	11.2
Mean intake energy drinks (g/day)	5.2	5.2	5.1	5.1	7.0	7.0	3.1	3.1	3.0	3.0
Relative standard error of estimate (%)	1.1	1.1	1.3	1.3	1.7	1.7	0.8	0.8	0.9	0.9
Lower 95% CI	3.0	3.0	2.4	2.4	3.5	3.5	1.5	1.5	1.2	1.2
Upper 95% CI	7.3	7.3	7.8	7.8	10.5	10.5	4.7	4.7	4.8	4.8
Mean intake fruit drink (g/day)	51.3	46.2	39.2	25.1	46.7	31.0	30.9	28.5	38.3	32.4
Relative standard error of estimate (%)	6.9	6.3	8.1	3.3	7.0	3.6	4.3	4.7	4.8	3.8
Lower 95% CI	37.4	33.6	23.0	18.4	32.7	23.7	22.3	19.0	28.8	24.8
Upper 95% CI	65.1	58.7	55.4	31.8	60.8	38.3	39.5	38.0	47.8	40.1
Mean intake cordial diluted (g/day)	54.5	20.2	55.7	32.3	38.7	29.7	31.5	16.5	26.1	15.4
Relative standard error of estimate (%)	8.3	4.3	9.7	5.0	7.8	6.4	7.3	3.1	5.1	3.4
Lower 95% CI	37.8	11.6	36.4	22.4	23.1	17.0	17.0	10.4	15.9	8.5
Upper 95% CI	71.2	28.7	75.0	42.3	54.3	42.5	46.1	22.6	36.3	22.3

	Population males									
Consumption of SSB	Males 2-9	10-14 yrs	15-19	20-24	25-34	35-44	45-54	55-64	65-74	75+
Mean intake carbonated drinks (g/day)	41.9	143.3	293.6	201.7	219.2	131.9	125.9	85.9	44.7	35.7
Relative standard error of estimate (%)	5.1	16.7	31.9	24.2	22.1	10.0	12.2	10.2	6.6	7.1
Lower 95% CI	31.8	109.9	229.8	153.2	174.9	111.9	101.4	65.4	31.4	21.4
Upper 95% CI	52.1	176.8	357.4	250.1	263.5	152.0	150.4	106.3	58.0	49.9
Mean intake sports drinks (g/day)	12.0	12.0	12.0	12.0	12.0	12.0	12.0	12.0	12.0	12.0
Relative standard error of estimate (%)	1.4	1.4	1.4	1.4	1.4	1.4	1.4	1.4	1.4	1.4
Lower 95% CI	9.2	9.2	9.2	9.2	9.2	9.2	9.2	9.2	9.2	9.2
Upper 95% CI	14.8	14.8	14.8	14.8	14.8	14.8	14.8	14.8	14.8	14.8
Mean intake energy drinks (g/day)	7.3	7.3	7.3	7.3	7.3	7.3	7.3	7.3	7.3	7.3
Relative standard error of estimate (%)	1.0	1.0	1.0	1.0	1.0	1.0	1.0	1.0	1.0	1.0
Lower 95% CI	5.4	5.4	5.4	5.4	5.4	5.4	5.4	5.4	5.4	5.4
Upper 95% CI	9.2	9.2	9.2	9.2	9.2	9.2	9.2	9.2	9.2	9.2
Mean intake fruit drink (g/day)	59.9	56.2	47.0	91.6	48.8	42.7	20.6	25.3	19.2	19.9
Relative standard error of estimate (%)	8.9	13.5	10.4	22.7	8.1	7.7	3.3	5.2	3.9	4.6
Lower 95% CI	42.1	29.1	26.1	46.1	32.5	27.2	14.0	14.8	11.3	10.7
Upper 95% CI	77.7	83.3	67.9	137.0	65.1	58.1	27.2	35.8	27.0	29.0
Mean intake cordial diluted (g/day)	51.8	76.0	40.2	55.9	42.3	41.4	55.0	12.4	16.7	30.6
Relative standard error of estimate (%)	8.2	15.1	9.2	18.5	11.5	9.6	12.5	2.9	4.4	8.7
Lower 95% CI	35.4	45.7	21.8	18.9	19.3	22.2	30.1	6.6	8.0	13.3
Upper 95% CI	68.2	106.3	58.6	92.9	65.3	60.6	80.0	18.3	25.4	48.0

	Population females									
Consumption of SSB	Females 2-9	10-14 yrs	15-19	20-24	25-34	35-44	45-54	55-64	65-74	75+
Mean intake carbonated drinks (g/day)	45.6	120.3	155.0	118.0	91.4	74.2	67.5	38.4	34.6	29.2
Relative standard error of estimate (%)	5.4	14.0	18.8	14.8	8.1	8.7	10.4	5.1	6.3	4.9
Lower 95% CI	34.8	92.3	117.3	88.4	75.1	56.8	46.7	28.2	22.0	19.4
Upper 95% CI	56.3	148.2	192.7	147.6	107.7	91.6	88.3	48.5	47.1	38.9
Mean intake sports drinks (g/day)	3.4	3.4	3.4	3.4	3.4	3.4	3.4	3.4	3.4	3.4
Relative standard error of estimate (%)	0.7	0.7	0.7	0.7	0.7	0.7	0.7	0.7	0.7	0.7
Lower 95% CI	1.9	1.9	1.9	1.9	1.9	1.9	1.9	1.9	1.9	1.9
Upper 95% CI	4.9	4.9	4.9	4.9	4.9	4.9	4.9	4.9	4.9	4.9
Mean intake energy drinks (g/day)	2.0	2.0	2.0	2.0	2.0	2.0	2.0	2.0	2.0	2.0
Relative standard error of estimate (%)	0.4	0.4	0.4	0.4	0.4	0.4	0.4	0.4	0.4	0.4
Lower 95% CI	1.1	1.1	1.1	1.1	1.1	1.1	1.1	1.1	1.1	1.1
Upper 95% CI	2.8	2.8	2.8	2.8	2.8	2.8	2.8	2.8	2.8	2.8
Mean intake fruit drink (g/day)	59.3	57.6	44.2	53.8	36.6	18.6	11.1	22.4	23.8	30.4
Relative standard error of estimate (%)	6.9	11.0	7.9	11.0	4.8	3.4	2.4	5.4	4.2	11.2
Lower 95% CI	45.5	35.6	28.3	31.7	27.0	11.8	6.3	11.6	15.3	7.9
Upper 95% CI	73.0	79.7	60.0	75.9	46.2	25.5	15.9	33.2	32.2	52.9
Mean intake cordial diluted (g/day)	36.9	34.0	31.9	17.4	29.8	21.8	13.7	15.9	14.3	5.2
Relative standard error of estimate (%)	6.8	8.4	11.8	4.9	5.7	4.0	2.8	4.2	4.1	1.6
Lower 95% CI	23.4	17.2	8.3	7.5	18.3	13.9	8.1	7.5	6.0	2.1
Upper 95% CI	50.4	50.7	55.4	27.2	41.3	29.7	19.3	24.3	22.5	8.4
