# Supplementary material for: Modelled health benefits of a sugar-sweetened beverage tax across different socioeconomic groups in Australia: A cost-effectiveness and equity analysis
Source: PLoS Med. 2017 Jun 27;14(6):e1002326. doi: 10.1371/journal.pmed.1002326 (PMC5486958; doi:10.1371/journal.pmed.1002326)
Supplement: S2 Table — (PDF) [file pmed.1002326.s007.pdf]

S2 Table. Impact of a 20% tax on demand for SSBs by SEIFA quintile and total population

% change demanded per capita	Quintile 1 (lowest)	Quintile 2	Quintile 3	Quintile 4	Quintile 5 (highest)	All households
Soft drinks (pop, soda) *	-15.05	-14.84	-14.92	-13.87	-13.10	-11.52
Cordial (concentrate)	-45.55	-40.86	-35.63	-32.58	-29.98	-33.23
Fruit drink	-3.08	-14.99	-36.61	-19.36	-11.82	-25.50

Notes: All non-zero effects significant at 10% level.

*Soft drinks include standard soft drinks, sports and energy drinks, flavoured bottled water
