# Supplementary material for: Modelled health benefits of a sugar-sweetened beverage tax across different socioeconomic groups in Australia: A cost-effectiveness and equity analysis
Source: PLoS Med. 2017 Jun 27;14(6):e1002326. doi: 10.1371/journal.pmed.1002326 (PMC5486958; doi:10.1371/journal.pmed.1002326)
Supplement: S3 Table — (PDF) [file pmed.1002326.s008.pdf]

S3 Table. Estimating the cost of sugar-sweetened beverage tax legislation

Variable	Value	Source or Method of calculation
Senate annual expenses	23,596,000	Average of annual expenses 2010-2015 Department of Senate Annual Reports[1]
House of Representatives annual expenses	25,800,000	Average of annual expenses 2010-2015. Department of House of Representatives Annual Reports[2]
Office of Parliamentary Counsel - legislation drafting and publication	15,690,000	Office of Parliamentary Counsel Budgets[3]
Annual parliamentarians' salaries	3,363,000	Number of hours spent on legislation/total working hours in a year = 18% times total parliamentarians salaries from Senate budget statements - resource statements Third Party Drawdowns on behalf of other departments[4]
Total annual cost of running the parliamentary service (PS)	65,086,000	Sum of variables above
Total annual cost of policy advice related to new laws provided by government agencies to parliament (PA)	39,443,000	$59,273,246 from Wilson et al [5] Converted to $AU 2010[6]
No. bills passed	170	Average of number of bills passed by Senate [7]
Regulatory impact statement (RIS)	$440,000	Australian Government cost of RIS[8]
Average cost of new legislation	$1,090,000	Total costs= PS+PA+RIS/no. bills

References

1. Parliament of Australia. Department of Senate Annual Reports Canberra, Australia: 2015

2. Parliament of Australia. Department of House of Representatives Annual Reports Canberra, Australia: 2015

3. Australian Government Attormey-General's Department. Budgets Canberra, Australia: 2015

4. Parliament of Australia. Portfolio budget statements Canberra, Australia 2015. Available from: http://www.aph.gov.au/About_Parliament/Parliamentary_Departments/Department_of_the_Senate/Portfolio_Budget_Statements.

5. Wilson N, Nghiem N, Foster R, et al. Estimating the cost of new public health legislation. *Bulletin of the World Health Organization*. 2012;90:477-556.

6. Organisation for Economic Co-operation and Development. PPPs and exchange rates 2016 [Accessed: 2016 8 June]. Available from: http://stats.oecd.org/Index.aspx?datasetcode=SNA_TABLE4.

7. Parliament of Australia. Time spent on consideration of legislation - 43rd Parliament Canberra, Australia 2013 [Accessed: 2016 8 June]. Available from: http://www.aph.gov.au/Parliamentary_Business/Statistics/Senate_StatsNet/legislation/timespent/2013.

8. Australian Government Productivity Commission. Regulatory Impact Analysis: Benchmarking Productivity Commission Research Report. Commonwealth of Australia, 2012
