# Supplementary material for: Modelled health benefits of a sugar-sweetened beverage tax across different socioeconomic groups in Australia: A cost-effectiveness and equity analysis
Source: PLoS Med. 2017 Jun 27;14(6):e1002326. doi: 10.1371/journal.pmed.1002326 (PMC5486958; doi:10.1371/journal.pmed.1002326)
Supplement: S4 Table — (PDF) [file pmed.1002326.s009.pdf]

S4 Table. Cost effectiveness results of the sensitivity analyses

	Quintile 1 (95%CI)	Quintile 2 (95%CI)	Quintile 3 (95%CI)	Quintile 4 (95%CI)	Quintile 5 (95%CI)	Total (95%CI)
**20% sugar-sweetened beverage tax including flavoured milk**						
Total HALYs saved over lifetime (thousands)	58.1 (18.0, 97.7)	54.9. (32.9, 76.7)	54.6 (22.9, 85.4)	34.9 (30.1, 40.6)	28.2 (12.2, 44.3)	192.7 (75.8, 301.2)
Total years of life saved over lifetime (thousands)	44.3 (13.6, 72.9)	41.0. (24.76.7)	41.1 (17.2, 63.8)	25.9 (23.0, 29.3)	21.7 (10.0, 33.6)	121.1 (47.8, 189.9)
Total healthcare cost savings over lifetime (millions of dollars)	473.0 (145.6, 767.5)	473.1 (286.5, 656.4)	476.2 (196.9, 738.6)	325.0 (273.6, 381.5)	260.3 (111.6, 407.7)	1,951.4 (1,687.2, 2,218.3)
Net cost per HALY saved	Cost saving	Cost saving	Cost saving	Cost saving	Cost saving	Cost saving
Out-of-pocket healthcare costs saved over lifetime (millions of dollars)	40.2 (34.8, 45.7)	40.7 (35.2,46.3)	57.8 (50.0, 65.8)	79.3 (68.6, 90.2)	121.4 (105.0, 138.0)	339.5 (293.6, 386.0)
Out-of-pocket costs of tax (dollars per person, yearly)	40.90 (24.80, 64.0)	37.30 (22.80, 57.70)	33.30 (19.50, 51.60)	30.60 (17.80, 49.40)	33.20 (20.50, 51.10)	34.10 (20.80, 53.40)
% expenditure food and non-alcoholic drinks	0.62%	0.40%	0.30%	0.25%	0.23%	0.32%
**50% pass-through of 20% sugar-sweetened beverage tax (10% tax rate)**						
Total HALYs saved over	29.2	27.3	27.1	16.5	14.7	89.0

lifetime (thousands)	(26.1, 32.6)	(4.6, 49.1)	(10.9, 43.7)	(14.1, 19.3)	(13.2, 16.4)	(33.6, 144.8)
Total years of life saved over lifetime (thousands)	22.1<br>(20.9, 23.4)	20.3<br>(3.4, 36.3)	20.5<br>(8.4, 32.7)	12.2<br>(10.8, 13.9)	11.2<br>(10.5, 12.0)	61.5<br>(23.8, 99.2)
Total healthcare cost savings over lifetime (millions of dollars)	238.5<br>(211.4, 265.0)	235.7<br>(40.3, 154.0)	236.8<br>(95.4, 376.9)	154.0<br>(129.8, 181.7)	133.7<br>(118.0, 151.3)	957.5<br>(829.8, 1,102)
Net cost per HALY saved	Cost saving	Cost saving	Cost saving	Cost saving	Cost saving	Cost saving
Out-of-pocket healthcare costs saved over lifetime (millions of dollars)	19.7<br>(17.1, 22.7)	20.0<br>(17.3,23.0)	28.4<br>(24.6, 32.7)	38.9<br>(33.7, 44.8)	59.6<br>(51.6, 68.6)	166.6<br>(144.4, 191.8)
Out-of-pocket costs of tax (dollars per person, yearly)	20.7<br>(12.40, 31.80)	17.10<br>(12.50, 22.60)	17.90<br>(11.30, 27.40)	15.70<br>(9.30, 25.10)	17.20<br>(10.60, 26.30)	16.90<br>(10.30, 25.80)
% expenditure food and non-alcoholic drinks	0.31%	0.18%	0.16%	0.13%	0.12%	0.32%
**30% sugar-sweetened beverage tax**						
Total years of life saved over lifetime (thousands)	70.5<br>(23.3, 112.6)	69.4<br>(47.1, 92.3)	66.2<br>(27.1, 100.0)	43.8<br>(37.5, 51.5)	38.5<br>(17.5, 58.0)	224.5<br>91.6, 346.0)
Total healthcare cost savings over lifetime (millions of dollars)	53.9<br>(17.9, 85.6)	51.9<br>(35.6, 68.1)	50.2<br>(20.3, 75.9)	32.6<br>(28.7, 37.0)	29.5<br>(13.6, 43.9)	155.5<br>(62.9, 238.7)
Net cost per HALY saved	574.4<br>(195.7, 919.7)	598.6<br>(405.8, 788.3)	576.1<br>(233.2, 865.6)	407.4<br>(341.1, 480.2)	352.3<br>(164.4, 536.2)	2501.4<br>(2,181.9, 2,820.4)
Out-of-pocket healthcare costs saved over lifetime	Cost saving	Cost saving	Cost saving	Cost saving	Cost saving	Cost saving

(millions of dollars)						
Out-of-pocket costs of tax (dollars per person, yearly)	**51.6 (6.1, 45.0)**	**52.2 (6.2, 45.5)**	**74.2 (8.8, 64.7)**	**101.7 (12.1,88.7)**	**155.6 (18.4,135.7)**	**435.2 (379.7, 490.8)**
% expenditure food and non-alcoholic drinks	51.40 (39.10, 67.90)	47.10 (36.10, 61.10)	49.60 (37.90, 62.70)	46.70 (38.30, 56.90)	**33.50** (**20.70**, 51.00)	51.20 (31.20, 80.10)
Total HALYs saved over lifetime (thousands)	0.78%	0.50%	0.45%	0.39%	0.23%	0.48%
50c per litre tax						
Total HALYs saved over lifetime (thousands)	30.4 (4.4, 54.7)	31.0 (4.0, 58.2)	33.2 (13.1, 51.3)	20.3 (17.5, 23.6)	12.7 (5.9, 19.5)	167.5 (148.5, 189.6)
Total years of life saved over lifetime (thousands)	23.1 3.4, 42.2	23.2 2.8, 43.3	25.1 10.0, 39.1	15.0 13.3, 16.9	9.7 4.5, 14.9	116.0 108.8, 122.9
Total healthcare cost savings over lifetime (millions of dollars)	248.2 34.8, 445.5	264.3 38.4, 486.3	289.1 117.3, 456.4	**188.9 159.5, 220.1**	116.4 53.2, 180.7	1,141.0 989.8, 1,309.7
Net cost per HALY saved	**23.5 (20.4, 27.0)**	**23.8 (20.6, 27.3)**	**33.8 (29.3, 38.8)**	**46.4 (40.2, 53.2)**	**71.0 (61.6, 81.5)**	**198.5 (172.2, 227.9)**
Out-of-pocket healthcare costs saved over lifetime (millions of dollars)	34.40 (31.90, 37.00)	27.80 (25.00, 30.90)	30.50 (26.80, 34.70)	23.60 (20.60, 26.70)	**25.70** (**23.70**, 27.90)	25.80 (23.80, 27.80)
Out-of-pocket costs of tax (dollars per person, yearly)	0.32%	0.42%	0.33%	0.21%	0.21%	0.18%
