# Supplementary material for: Correction: Modelled health benefits of a sugar-sweetened beverage tax across different socioeconomic groups in Australia: A cost-effectiveness and equity analysis
Source: PLoS Med. 2020 Jul 29;17(7):e1003310. doi: 10.1371/journal.pmed.1003310 (PMC7390261; doi:10.1371/journal.pmed.1003310)
Supplement: S4 Table — (PDF) [file pmed.1003310.s001.pdf]

**S4 Table. Cost effectiveness results of the sensitivity analyses**

| | Quintile 1 (95%CI) | Quintile 2 (95%CI) | Quintile 3 (95%CI) | Quintile 4 (95%CI) | Quintile 5 (95%CI) | Total (95%CI) |
|---|---|---|---|---|---|---|
| **20% sugar-sweetened beverage tax including flavoured milk** | | | | | | |
| Total HALYs saved over lifetime (thousands) | 58.1 (18.0, 97.7) | 54.9. (32.9, 76.7) | 54.6 (22.9, 85.4) | 34.9 (30.1, 40.6) | 28.2 (12.2, 44.3) | 192.7 (75.8, 301.2) |
| Total years of life saved over lifetime (thousands) | 44.3 (13.6, 72.9) | 41.0. (24.76.7 ) | 41.1 (17.2, 63.8) | 25.9 (23.0, 29.3) | 21.7 (10.0, 33.6) | 121.1 (47.8, 189.9) |
| Total healthcare cost savings over lifetime (millions of dollars) | 473.0 (145.6, 767.5) | 473.1 (286.5, 656.4) | 476.2 (196.9, 738.6) | 325.0 (273.6, 381.5) | 260.3 (111.6, 407.7) | 1,951.4 (1,687.2, 2,218.3) |
| Net cost per HALY saved | Cost saving | Cost saving | Cost saving | Cost saving | Cost saving | Cost saving |
| Out-of-pocket healthcare costs saved over lifetime (millions of dollars) | 40.2 (34.8, 45.7) | 40.7 (35.2,46.3) | 57.8 (50.0, 65.8) | 79.3 (68.6, 90.2) | 121.4 (105.0, 138.0) | 339.5 (293.6, 386.0) |
| Out-of-pocket costs of tax (dollars per person, yearly) | 40.90 (24.80, 64.0) | 37.30 (22.80, 57.70) | 33.30 (19.50, 51.60) | 30.60 (17.80, 49.40) | 33.20 (20.50, 51.10) | 34.10 (20.80, 53.40) |
| % expenditure food and non-alcoholic drinks | 0.62% | 0.40% | 0.30% | 0.25% | 0.23% | 0.32% |
| **50% pass-through of 20% sugar-sweetened beverage tax (10% tax rate)** | | | | | | |
| Total HALYs saved over | 29.2 | 27.3 | 27.1 | 16.5 | 14.7 | 89.0 |

| | | | | | | |
|---|---|---|---|---|---|---|
| lifetime (thousands) | (26.1, 32.6) | (4.6, 49.1) | (10.9, 43.7) | (14.1, 19.3) | (13.2, 16.4) | (33.6, 144.8) |
| Total years of life saved over lifetime (thousands) | 22.1<br>(20.9, 23.4) | 20.3<br>(3.4, 36.3) | 20.5<br>(8.4, 32.7) | 12.2<br>(10.8, 13.9) | 11.2<br>(10.5, 12.0) | 61.5<br>(23.8, 99.2) |
| Total healthcare cost savings over lifetime (millions of dollars) | 238.5<br>(211.4, 265.0) | 235.7<br>(40.3, 154.0) | 236.8<br>(95.4, 376.9) | 154.0<br>(129.8, 181.7) | 133.7<br>(118.0, 151.3) | 957.5<br>(829.8, 1,102) |
| Net cost per HALY saved | Cost saving | Cost saving | Cost saving | Cost saving | Cost saving | Cost saving |
| Out-of-pocket healthcare costs saved over lifetime (millions of dollars) | 19.7<br>(17.1, 22.7) | 20.0<br>(17.3,23.0) | 28.4<br>(24.6, 32.7) | 38.9<br>(33.7, 44.8) | 59.6<br>(51.6, 68.6) | 166.6<br>(144.4, 191.8) |
| Out-of-pocket costs of tax (dollars per person, yearly) | 20.7<br>(12.40, 31.80) | 17.10<br>(12.50, 22.60) | 17.90<br>(11.30, 27.40) | 15.70<br>(9.30, 25.10) | 17.20<br>(10.60, 26.30) | 16.90<br>(10.30, 25.80) |
| % expenditure food and non-alcoholic drinks | 0.31% | 0.18% | 0.16% | 0.13% | 0.12% | 0.32% |
| **30% sugar-sweetened beverage tax** | | | | | | |
| Total HALYs saved over lifetime (thousands) | 70.5<br>(23.3, 112.6) | 69.4<br>(47.1, 92.3) | 66.2<br>(27.1, 100.0) | 43.8<br>(37.5, 51.5) | 38.5<br>(17.5, 58.0) | 224.5<br>91.6, 346.0) |
| Total years of life saved over lifetime (thousands) | 53.9<br>(17.9, 85.6) | 51.9<br>(35.6, 68.1) | 50.2<br>(20.3, 75.9) | 32.6<br>(28.7, 37.0) | 29.5<br>(13.6, 43.9) | 155.5<br>(62.9, 238.7) |
| Total healthcare cost savings over lifetime (millions of dollars) | 574.4<br>(195.7, 919.7) | 598.6<br>(405.8, 788.3) | 576.1<br>(233.2, 865.6) | 407.4<br>(341.1, 480.2) | 352.3<br>(164.4, 536.2) | 2501.4<br>(2,181.9, 2,820.4) |
| Net cost per HALY saved | Cost saving | Cost saving | Cost saving | Cost saving | Cost saving | Cost saving |

| | | | | | | |
|---|---|---|---|---|---|---|
| Out-of-pocket healthcare costs saved over lifetime (millions of dollars) | **51.6 (6.1, 45.0)** | **52.2 (6.2, 45.5)** | **74.2 (8.8, 64.7)** | **101.7 (12.1,88.7)** | **155.6 (18.4,135.7)** | **435.2 (379.7, 490.8)** |
| Out-of-pocket costs of tax (dollars per person, yearly) | 51.40 (39.10, 67.90) | 47.10 (36.10, 61.10) | 49.60 (37.90, 62.70) | 46.70 (38.30, 56.90) | **33.50** (**20.70**, 51.00) | 51.20 (31.20, 80.10) |
| % expenditure food and non-alcoholic drinks | 0.78% | 0.50% | 0.45% | 0.39% | 0.23% | 0.48% |
| 50c per litre tax | | | | | | |
| Total HALYs saved over lifetime (thousands) | 30.4 (4.4, 54.7) | 31.0 (4.0, 58.2) | 33.2 (13.1, 51.3) | 20.3 (17.5, 23.6) | 12.7 (5.9, 19.5) | 167.5 (148.5, 189.6) |
| Total years of life saved over lifetime (thousands) | 23.1 3.4, 42.2 | 23.2 2.8, 43.3 | 25.1 10.0, 39.1 | 15.0 13.3, 16.9 | 9.7 4.5, 14.9 | 116.0 108.8, 122.9 |
| Total healthcare cost savings over lifetime (millions of dollars) | 248.2 34.8, 445.5 | 264.3 38.4, 486.3 | 289.1 117.3, 456.4 | **188.9 159.5, 220.1** | 116.4 53.2, 180.7 | 1,141.0 989.8, 1,309.7 |
| Net cost per HALY saved | Cost saving | Cost saving | Cost saving | Cost saving | Cost saving | Cost saving |
| Out-of-pocket healthcare costs saved over lifetime (millions of dollars) | **23.5 (20.4, 27.0)** | **23.8 (20.6, 27.3)** | **33.8 (29.3, 38.8)** | **46.4 (40.2, 53.2)** | **71.0 (61.6, 81.5)** | **198.5 (172.2, 227.9)** |
| Out-of-pocket costs of tax (dollars per person, yearly) | 34.40 (31.90, 37.00) | 27.80 (25.00, 30.90) | 30.50 (26.80, 34.70) | 23.60 (20.60, 26.70) | **25.70** (**23.70**, 27.90) | 25.80 (23.80, 27.80) |
| % expenditure food and non-alcoholic drinks | 0.32% | 0.42% | 0.33% | 0.21% | 0.21% | 0.18% |
